# Synchrotron-based ν-XRF mapping and μ-FTIR microscopy enable to look into the fate and effects of tattoo pigments in human skin

**DOI:** 10.1038/s41598-017-11721-z

**Published:** 2017-09-12

**Authors:** Ines Schreiver, Bernhard Hesse, Christian Seim, Hiram Castillo-Michel, Julie Villanova, Peter Laux, Nadine Dreiack, Randolf Penning, Remi Tucoulou, Marine Cotte, Andreas Luch

**Affiliations:** 1 German Federal Institute for Risk Assessment (BfR), Department of Chemical and Product Safety, Max-Dohrn-Strasse 8-10, 10589 Berlin, Germany; 2 European Synchrotron Radiation Facility (ESRF), 38043 Grenoble, Cedex 9 France; 3 Physikalisch-Technische Bundesanstalt, Department of X-ray Spectrometry, Abbestrasse 2-12, 10587 Berlin, Germany; 4 Technische Universität Berlin, Institute for Optics and Atomic Physics, Hardenbergstrasse 36, 10623 Berlin, Germany; 5 Institute of Forensic Medicine, Ludwig-Maximilians University, Munich, Germany

## Abstract

The increasing prevalence of tattoos provoked safety concerns with respect to particle distribution and effects inside the human body. We used skin and lymphatic tissues from human corpses to address local biokinetics by means of synchrotron X-ray fluorescence (XRF) techniques at both the micro (μ) and nano (ν) scale. Additional advanced mass spectrometry-based methodology enabled to demonstrate simultaneous transport of organic pigments, heavy metals and titanium dioxide from skin to regional lymph nodes. Among these compounds, organic pigments displayed the broadest size range with smallest species preferentially reaching the lymph nodes. Using synchrotron μ-FTIR analysis we were also able to detect ultrastructural changes of the tissue adjacent to tattoo particles through altered amide I α-helix to β-sheet protein ratios and elevated lipid contents. Altogether we report strong evidence for both migration and long-term deposition of toxic elements and tattoo pigments as well as for conformational alterations of biomolecules that likely contribute to cutaneous inflammation and other adversities upon tattooing.

## Introduction

In recent years, the seemingly unstoppable trend for tattoos has brought safety concerns into the spotlight^1^. Currently, basic toxicological aspects, from biokinetics to possible alterations of the pigments, are largely uncertain. The animal experiments which would be necessary to address these toxicological issues were rated unethical because tattoos are applied as a matter of choice and lack medical necessity, similar to cosmetics^2^. Consequently, the hazards that potentially derive from tattoos were as yet only investigated by chemical analysis of the inks and their degradation products *in vitro* ^3–6^. Even though toxicological data might be available for some ink ingredients individually, information on *in vivo* interactions of the ink’s components and their fate within the body is rare.

**Figure 1:**
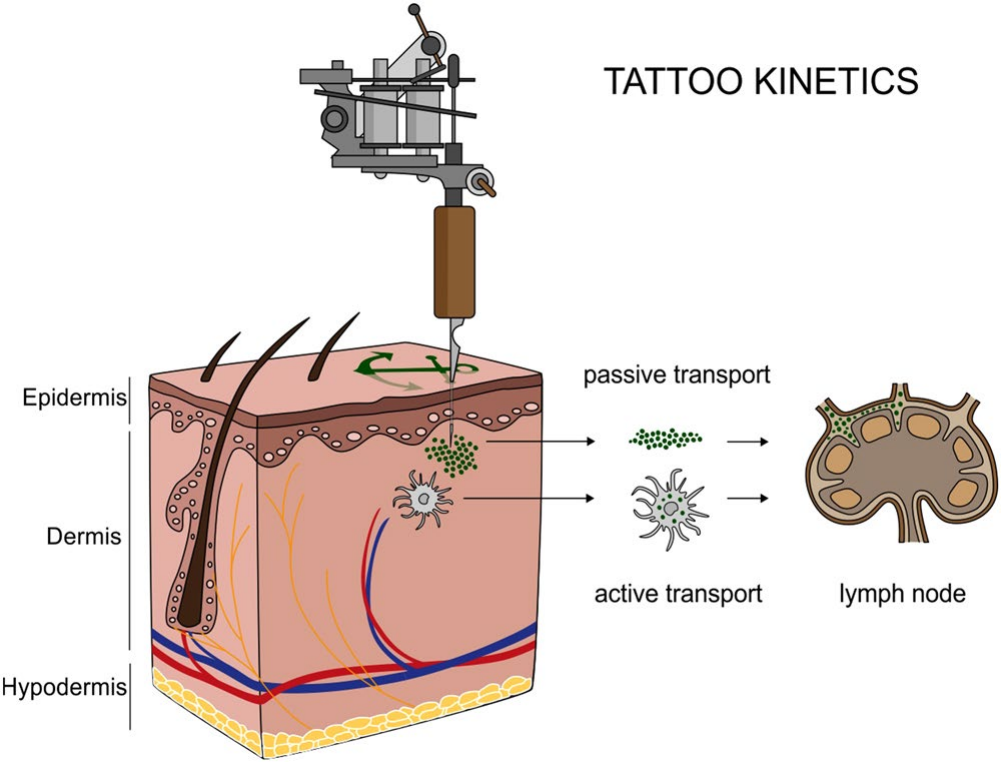
Translocation of tattoo particles from skin to lymph nodes. Upon injection of tattoo inks, particles can be either passively transported via blood and lymph fluids or phagocytized by immune cells and subsequently deposited in regional lymph nodes. After healing, particles are present in the dermis and in the sinusoids of the draining lymph nodes. The picture was drawn by the authors (i.e., C.S.).

Tattoos and permanent make-up work by depositing insoluble pigments into the dermal skin layer (Fig. 1). In conjunction with tattoos, pigmented and enlarged lymph nodes have been noticed in tattooed individuals for decades^7^. After the traumatic insertion of inks during the tattooing procedure, the body will excrete as many components as possible via the damaged epidermis. Other ways to clean the site of entrance are through active transport to lymph nodes by phagocytizing cells, or passively along the lymphatic vessels^8–11^. In addition to observations in humans, an *in vivo* study in mice revealed colored lymph nodes after tattooing with an azo pigment^12^. Even though this leaves little doubt that the pigment originates from corresponding tattoos, the origin and fate of pigments in human lymph nodes have never been analytically investigated so far. Lately, the only study analyzing human lymph nodes in tattooed individuals assessed its contents on carcinogenic polycyclic aromatic hydrocarbons deriving from carbon black particles^13^.

Tattoo pigments consist of either inorganic colorful metals and its oxides, or of polyaromatic compounds, all of which are thought to be biologically inert. It can thus be expected to find a broad range of elements in tattooed human tissue—among them the sensitizers nickel (Ni), chromium (Cr), manganese (Mn), and cobalt (Co)—as parts of color-giving pigments or element contamination^14–17^. Besides carbon black, the second most common used ingredient of tattoo inks is titanium dioxide (TiO_2_), a white pigment usually applied to create certain shades when mixed with colorants. The toxicity of TiO_2_ depends on its speciation (crystal structure) which can be either rutile or the more harmful photocatalytically active anatase^18^. The latter can lead to the formation of reactive oxygen species when exposed to sunlight. Delayed healing is thus often associated with white tattoos, along with skin elevation and itching^19^.

The contribution of tattoo inks to the overall body load on toxic elements, the speciation of TiO_2_, and the identities and size ranges of pigment particles migrating from subepidermal skin layers towards lymph nodes have never been analytically investigated in humans before. The average particle size in tattoo inks may vary from <100 nm to >1 µm^20^. Therefore most tattoo inks contain at least a small fraction of particles in the nano range.

Here, we analyzed tattooed human skin and regional lymph nodes originating from four donors (corpses). Inductively coupled plasma mass spectrometry (ICP-MS) was used to assess the general contents of elements in both tissues and tattoo inks after microwave digestion. Laser-desorption/ionization time-of-flight (LDI-ToF) MS facilitated the identification of organic pigments in enzyme-lysed samples. To precisely locate the different elements in the cutaneous and lymphatic microenvironments, to provide information on TiO_2_ speciation and to assess primary particle sizes at the nanometric scale in particle mixtures, however, synchrotron-based X-ray fluorescence investigations have been performed at both the micro (μ-XRF) and nano (ν-XRF) range. Furthermore, we assessed biomolecular changes in the respective tissues at the micrometric scale and in the proximity of tattoo particles using synchrotron-based Fourier transform infrared (μ-FTIR) spectroscopy.

## Results

### Organic pigments translocate from skin to lymph nodes

**Figure 2:**
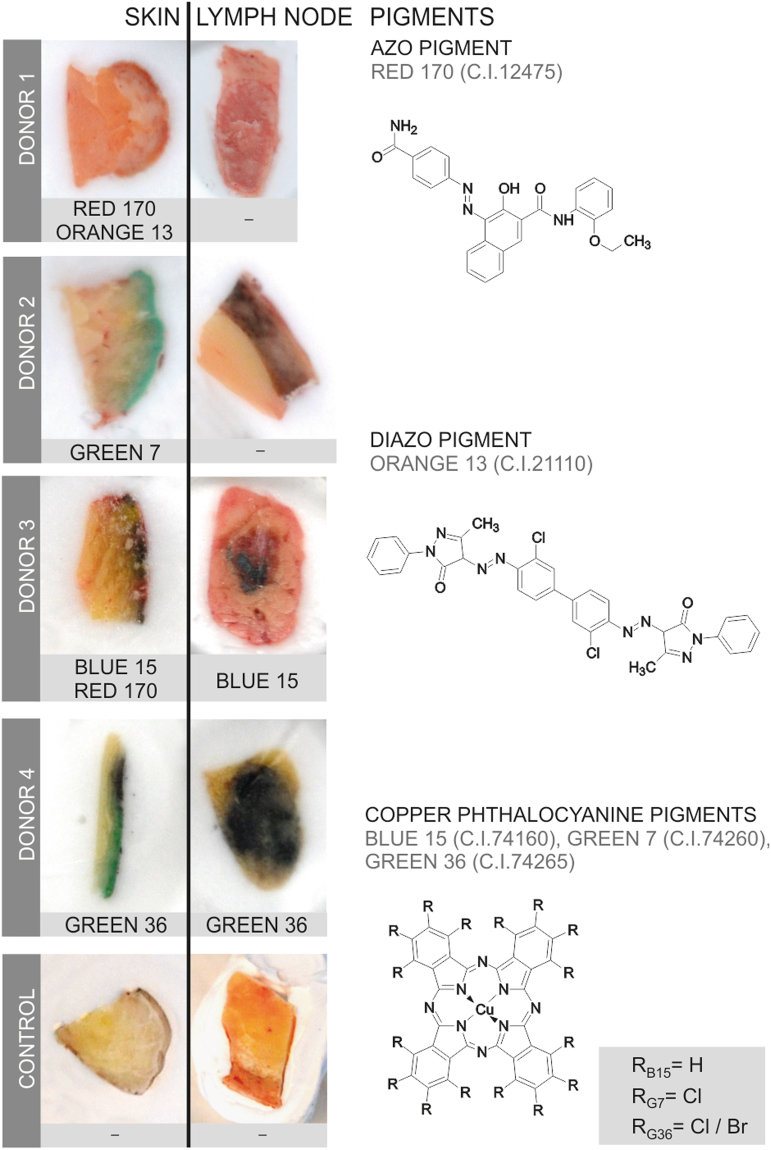
Organic pigments translocate from skin to lymph nodes. Organic pigments in lysed skin and lymph nodes were identified by means of LDI-ToF-MS. Adjacent skin and lymph tissue specimens (about 5–10 mm) are displayed in cryo-matrix after preparing thin sections for μ-FTIR and μ-XRF analyses. Skin specimens are oriented with its surface on the right side. Identified organic pigments are indicated below each sample. Chemical structures of the organic pigments identified in the samples are displayed on the right.

Providing analytical evidence of tattoo particles being distributed inside the human body was a key objective of this investigation. To this end, tissue samples of four individuals tattooed with orange, red, green or black and two non-tattooed control donors were analyzed for the presence of organic pigments. Detection of the same pigment species in both skin and regional lymph nodes revealed the drainage of tattoo particles in two out of four tattooed donors (Fig. 2).

Identification of organic pigments using LDI-ToF-MS has mostly been described using inks^21–23^. This technique is mainly based on isotope distributions and the molecular mass (see Supplementary Fig. S1). In the lysed tissues presented here, color-giving pigments were found to be copper phthalocyanines with either hydrogen, chlorine or bromine residues in three out of four skin samples. Reddish parts of the tattoos contained the azo group-containing pigments red 170 and orange 13 (Fig. 2).

For donors 1 and 2, the absence of organic pigments in the lymph nodes suggests either concentrations below the limit of detection (approx. 0.1–1% w/w pigment per extract), possible metabolic decomposition or drainage to alternative lymph nodes. The general ability for azo pigment translocation to lymph nodes was proven in additional skin and lymph node samples of donor 2 (Supplementary Table 1). On the other hand, carbon black particles possibly responsible for the black color in skin and lymph nodes (Fig. 2) were not accessible with the analytical methods used in this investigation. No xenobiotic pigment particles were detected in either skin or lymph tissue of the control samples.

### Tattoos contribute to the elemental load of lymph nodes

A central aim of this study was to assess to what extent tattooing increases the proportion of toxic elements in the body. We found Al, Cr, Fe, Ni and Cu quantitatively elevated in skin and lymph node specimens using ICP-MS analysis (Table 1 and Supplementary Table S2). For donor 4, Cd and Hg concentrations were found increased only in the lymph nodes, but not in the analyzed skin sections. These elements probably result from other tattoos that were not part of this study or other routes of exposure drained through the same lymphatic tissue. Non-quantitative evaluation of the survey scans revealed the presence of Ti, presumably derived from TiO_2_, in all tattooed skin samples but not in controls.

**Table 1:** Element concentrations per tissue weight (ppm) in human skin and lymph node samples analyzed by ICP-MS.

Donor	Tissue	Location	Al	Cr	Fe	Ni	Cu	Cd	other^#^
1	Skin	dorsal	0.92	0.74	64.7	0.59	2.51	0.15	**Ti**
	LN	left axillary	1.97	0.43	125	0.28	2.98	0.35	Zn, Rb
2	Skin	right leg	7.29	5.54	51.1	2.51	18.8	0.23	**Ti**
	LN	right inguinal	9.06	22.5	235	10.1	118	1.23	**Ti**, Mn
3	Skin	right arm	3.39	2.73	84.7	1.61	67.5	0.17	**Ti**, I
	LN	right axillary	5.08	13.9	221	6.74	28.7	0.28	**Ti**, Mn, Zn, Rb, I
4	Skin	left arm	15.4	4.07	120	0.45	199	0.52	**Ti**, **Br**
	LN	left axillary	4.16	0.67	138	0.30	15.3	146	**Ti**, **Br**, **Ba**, Mn, W, Rb, Hg
Control 1	Skin	proximal	0.75	0.16	35.6	0.08	1.44	0.12	Pb
	LN	axillary	1.11	0.31	64.4	1.09	12.9	0.47	
Control 2	Skin	proximal	0.76	0.60	37.6	0.15	1.41	0.25	
	LN	axillary	0.24	0.14	74.7	0.09	2.48	0.83	Zn, Rb
Literature values	Skin						0.35/0.42^a^	0.05/0.02^a^	
							.	.	
	LN		2000^c^	8.2^c^	1800^c^	0.28^b^ 3.7^c^	2.94/ 5.89^a^ 7.6^c^	0.24/ 0.20^a^ 2.5^c^	

Abbreviations: LN = lymph node. Elements measured (non-specified oxidation states): aluminum (Al), barium (Ba), bromine (Br), cadmium (Cd), chromium (Cr), copper (Cu), iodine (I), iron (Fe), lead (Pd), manganese (Mn), mercury (Hg), nickel (Ni), rubidium (Rb), titanium (Ti), tungsten (W), and zinc (Zn).

^#^Non-quantitatively identified elements (elements marked in bold are associated with tattoo pigments).

^a^Wet basis, average from 21 male/female cadavers^24^.

^b^Tissue dry weight, average in hilar lymph nodes from 3 cadavers^29^.

^c^Tissue dry weight, average in hilar lymph nodes from 12 male cadavers^32^.

The microwave digestion used in this investigation is not suitable to fully dissolve Fe and Ti, although no residual particles were visible. Therefore Fe concentrations might not represent the total amount in the samples, but they enable the distinction between physiological concentrations in controls and samples containing extrinsic Fe. The elevated levels of Fe found in the skin and lymph nodes of donor 4 imply an additional use of iron-based pigments. In donors 1, 2 and 3, Fe concentrations were only increased in adjacent lymph nodes and not in the corresponding skin samples (Table 1). Fe concentrations can also be affected by residual blood within the tissue samples.

In donor 4, the use of pigment copper phthalocyanine green 36, as identified with LDI-ToF-MS, is reflected by high amounts of Cu in skin and lymph nodes as well as the non-quantitative detection of Br (Table 1). By contrast, although pigment copper phthalocyanine green 7 was well detectable with our LDI-ToF-MS approach in the skin of donor 2, it was not in the corresponding regional lymph node. Increased Cu levels measured by ICP-MS in this adjacent sample, however, suggest the presence of this copper phthalocyanine pigment. In light of the other two copper phthalocyanines applied in donor 2 (green 7) and 3 (blue 15) elevated Cu levels in skin came without surprise (Table 1). In donor 2, Cu levels in lymph nodes are strongly increased despite the fact that green 7 could not be detected with LDI-ToF-MS. However, adjacent samples of tissue were used for each analysis. Given the nature of the samples, pigment deposition within skin and lymph nodes is not homogeneous and therefore explaining the different findings. Interestingly, the non-tattooed control donor 1 also had slightly elevated levels of about 13 ppm Cu in the lymph nodes which is still in the range of the average 5.89 ± 8.03 ppm of Cu detectable in lymph nodes of female cadavers (Table 1)^24^.

Additionally, Ni and Cr were found in the human specimens. Since Ni levels were increased in the skin and lymph nodes of donor 2 and 3, the likely source is the tattoo. In different studies, both elements were linked to adverse reactions occurring in tattooed patients^25–28^. Ni and Cr are known to be allergenic as well as carcinogenic. Ni concentrations of 0.28–10.05 ppm total tissue weight found here are within the range of 0.8–3.7 ppm dry weight Ni in hilar lymph nodes in previous studies^29^. Cd was drastically elevated only in the lymph node of donor 4. For all other samples, Cd tissue burdens lie within normal values^24^.

Finally, Al was also present in skin and lymph node tissues of the three tattooed donors 2, 3 and 4 (Table 1). Since auxiliary lymph nodes have been investigated in the case of donor 2 and 3, co-exposure from antiperspirants containing various aluminum salts cannot be excluded, neither in tattooed nor control samples. However, Al concentrations in the controls were lower. The light metal Al has recently attracted attention because of its accumulation in breast cancer tissue^30^. While its role in the emerging of neoplasia is currently highly disputed, its contribution to the occurrence of hypersensitivity granulomas associated with tattoos has been proven since decades^31^.

### μ-XRF mapping links metallic elements to tattoo particles

**Figure 3:**
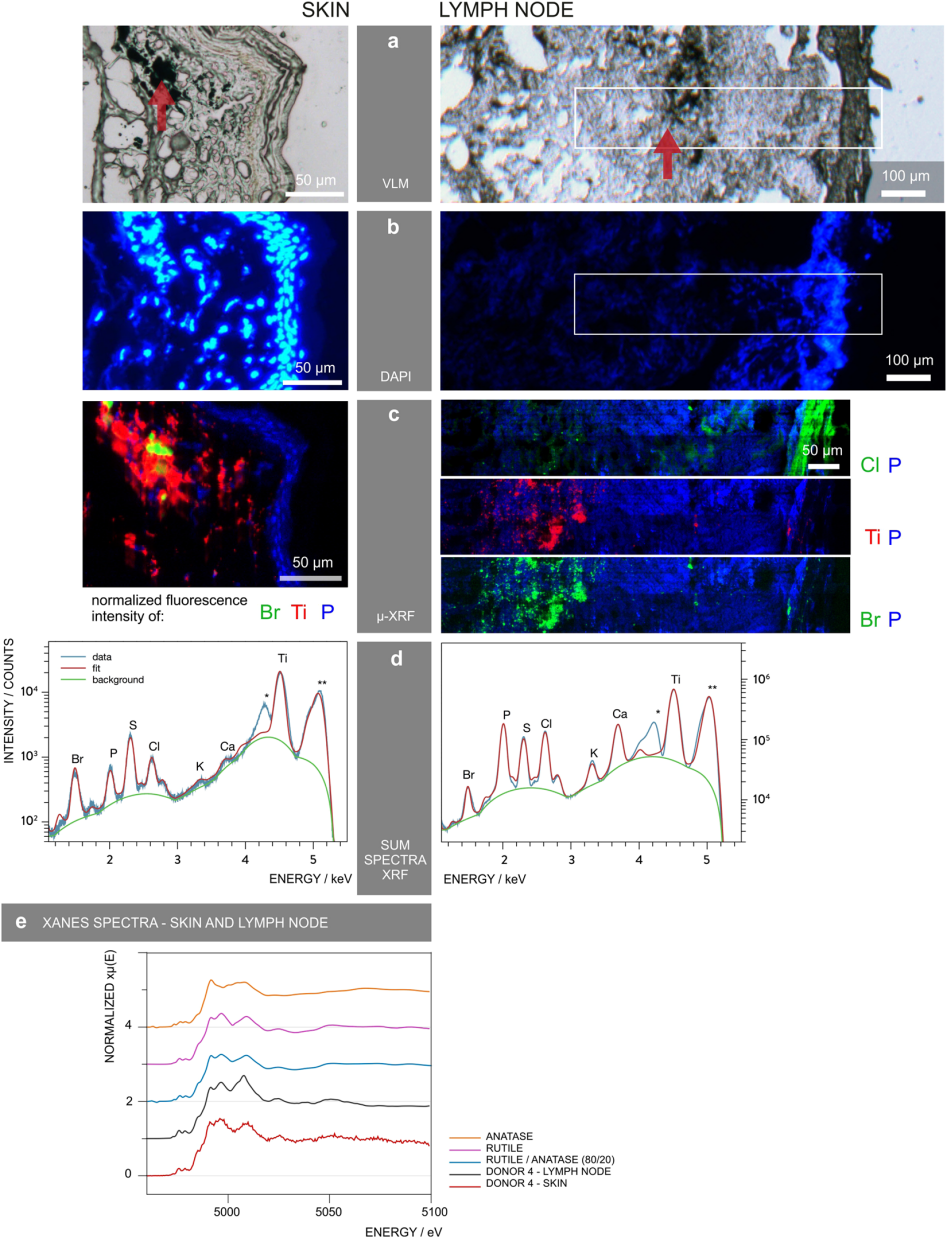
μ-XRF mapping identifies and locates tattoo particle elements in skin and lymph node tissue sections. Sections of skin and lymph node tissue from donor 4 were analyzed by means of synchrotron μ-XRF. (**a**) Visible light microscopy (VLM) images of the area mapped by μ-XRF. Tattoo pigments are indicated by a red arrow. (**b**) DAPI staining of adjacent sections showing the cell nuclei. (**c**) μ-XRF maps of P, Ti, Cl and/or Br. For the lymph node, areas of similar size are marked in (**a**) and (**b**). (**d**) Average μ-XRF spectra over the full area displayed in (**c**) *diffraction peak from sample support; **scatter peak of the incoming beam. (**e**) Ti K-edge μ-XANES spectra of skin and lymph node compared to transmission XANES spectra of reference material of rutile, anatase and an 80/20 rutile/anatase mixture calculation.

In order to link elements found with ICP-MS in tattoo pigment particles and to locate them inside the tissues, μ-XRF imaging was carried out with sub-micrometric probes over skin and lymph node sections (Fig. 3a–d). The location of particles can be altered by sample preparation. Since transversal sections were made by moving the knife parallel to the skin surface, the depth profile of the pigments should remain unaffected. Thin sections of skin and lymph nodes from donors 1, 3 and 4 were analyzed at the ESRF beamline ID21, with an exciting energy of 5.05 keV (Fig. 3a–d and Supplementary Fig. S2). Since the thin sections were deposited on BaF_2_ windows for further μ-FTIR analyses, the energy was chosen to avoid excitation of Ba L-lines (<5.24 keV). Results of donor 4 are displayed in Fig. 3 as an example.

The majority of particles in the skin tissue were surrounded by phosphor-rich nuclei visualized by DAPI staining in fluorescence light microscopy (Fig. 3b) and integration of the element P in μ-XRF analysis (Fig. 3c). It was previously shown that tattoo particles can primarily be found around vessels^10^ which might account for the high cell density in the dermis co-localized with the pigments.

Intensities of Ti K-lines and Br L-lines were extracted to map the distribution of TiO_2_ and the highly brominated pigment copper phthalocyanine green 36 (Fig. 3c). Since the Br L-lines completely overlap with the Al K-lines, both may contribute to the intensity of the peak. However, LDI-ToF-MS analysis revealed the presence of pigment green 36 (Fig. 2) and the following ν-XRF results from ID16B acquired at 17.5 keV, i.e. above Br K-edge (13.47 keV) undermined the primarily Br-related contribution (see Supplementary Fig. S3).

Tattoo particles containing Ti and Br are adjacent to each other with only a slight overlap in skin and seem to be more evenly co-localized in lymph tissue (Fig. 3c). Both elements were found in the dermis of donor 4 directly beneath the cell nuclei-rich epidermis and up to a few hundred micrometers deep in the skin. In the lymph nodes, some particles were deposited in the stroma directly beneath the capsule. The bulk of Ti and Br containing particles, however, became visible as pigment agglomerates at a distance of about 250 µm to the lymph node capsule. Conversely, Cl concentrations are highest in the lymph node capsule and lower concentrations can be found in the particle region as part of the pigment phthalocyanine green 36.

All analyzed samples from the tattooed donors contained Ti. It is unlikely that other sources, e.g. sun screens, would explain the high amounts found in this investigation. Elevated amounts of Ti are only expected in lung and hilar lymph nodes from respiratory exposure^32^. Other highly abundant elements are K and Ca as they are physiologically present in cells (Fig. 3d).

We also investigated if the Ti present is the expected white pigment TiO_2_ and whether the stable rutile and/or the more photoreactive anatase crystal phases were used in tattoo inks. Micro X-ray absorption near edge structure (μ-XANES) spectra at the Ti K-edge were collected for the skin and lymph nodes of donors 1, 3 and 4. The spectra of donor 4 showed more qualitative correlation with the reference spectrum of rutile than with that of anatase (Fig. 3e). A clear switch of peak maxima between 4.99–5 keV occurs as a difference of both types of crystal structures. A calculated spectrum of 20% anatase and 80% rutile mixture is not clearly distinguishable from pure rutile, but shows a pre-edge at around 4.97 keV, similar to the μ-XANES spectra of the tattooed samples. Therefore, mostly rutile TiO_2_ is present in all tattooed donors, with minor amounts of anatase (Fig. 3e and Supplementary Fig. S2).

### Particle size varies between pigment species

**Figure 4:**
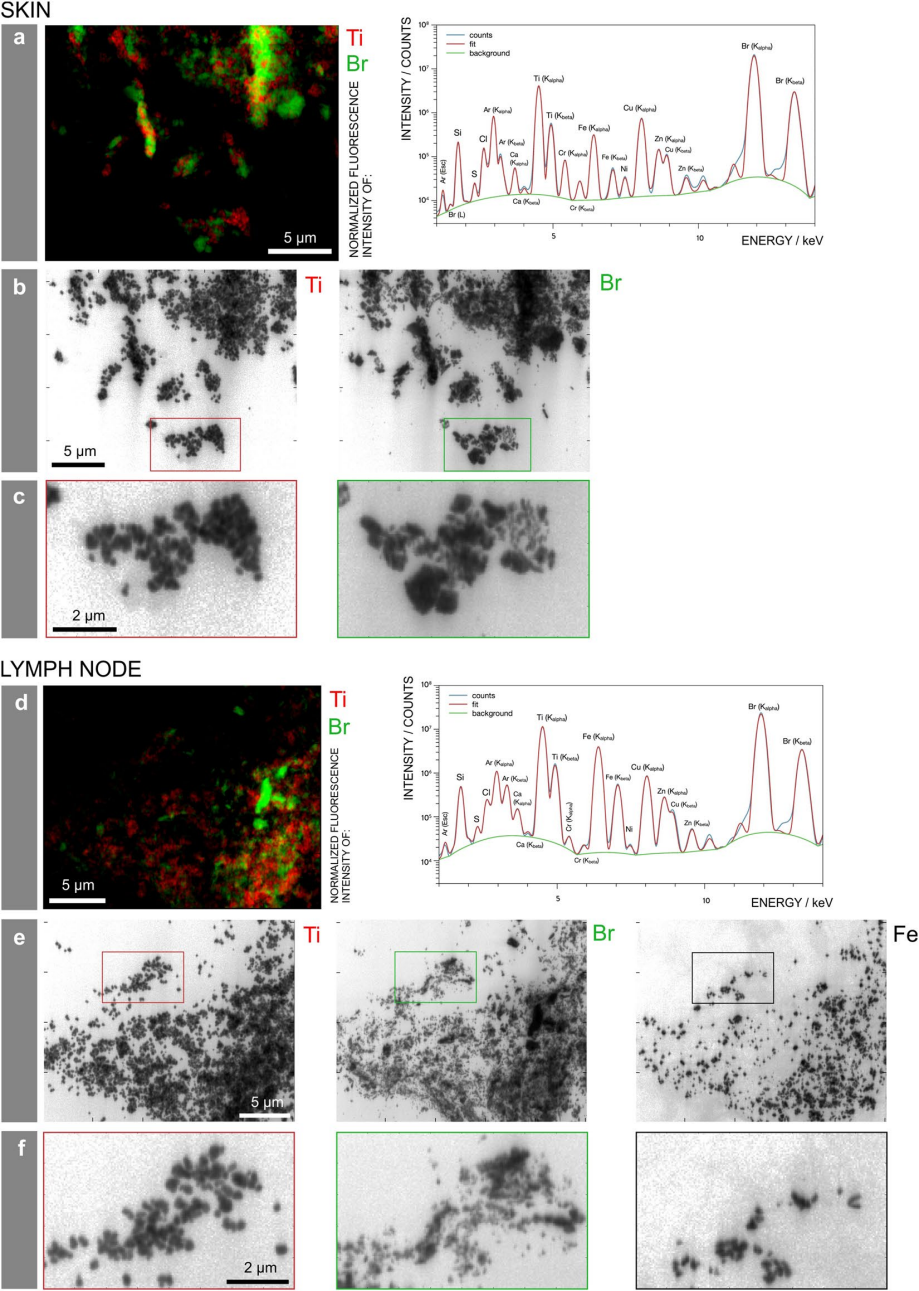
Particle mapping and size distribution of different tattoo pigment elements. Skin and lymph node of donor 4 were analyzed by means of synchrotron ν-XRF. (**a**,**d**) Ti and the Br containing pigment phthalocyanine green 36 are located next to each other. Average XRF spectra over the full area displayed in the regions of interest reveal the presence of Br, Si, S, Cl, Ca, Ti, Cr, Fe, Ni, Cu, and Zn. (**b**,**e**) Log scale mappings of Ti, Br and Fe in the same areas as displayed in (**a)** and (**d**) reveal primary particle sizes of different pigment species. (**c**,**f**) Magnifications of the indicated areas in (**b**) and (**e**), respectively.

The obtained μ-XRF maps of skin and lymph node sections show large tattoo particle agglomerates up to several micrometers (Fig. 3c). However, it is known that small-sized particles are preferentially transported to lymph nodes. The 0.3 × 0.7 µm² beam size for μ-XRF mapping at ID21 was therefore a limiting factor for the determination of particle sizes. To assess the primary particle sizes, we additionally performed ν-XRF investigations by applying a beam of 50 × 50 nm² at 17.5 keV in order to excite the Br K-lines. Experiments were carried out in adjacent sections of skin and lymph node from donor 4, prepared on ultralene foil (Fig. 4). We detected three different pigment particles, each showing a different elemental composition and distribution within the same area (Fig. 4b,e). The average particle size of TiO_2_ in both skin and lymph nodes was 180 nm with a standard deviation of 23 nm and a standard error of 7 nm. Therefore this rather large particle size does not prevent distribution via the lymph fluid.

In contrast, the pigment phthalocyanine green 36 analyzed by ν-XRF mapping of Br was much more polydisperse, with particles presumably smaller than the resolution of 50 nm and up to the µm range in skin. In lymph node tissue, particles containing Br were smaller, with fewer particles of a larger size (Fig. 4c,f). Hence it can be assumed that the transport of smaller particles is preferential.

With the chosen energy, Br can be unequivocally identified from its K-lines emission. The skin and lymph node of donor 4 also contained Cu, related to the identified copper phthalocyanine pigments, and its maps show perfect co-localization with Br (see Supplementary Fig. S3). Additionally, Fe particles were present in the lymph node but not skin tissue and therefore possibly originate from another tattoo or route of exposure (Fig. 4c,f and Supplementary Fig. S3).

### Tattoo particles induce biomolecular changes

The synchrotron-based μ-FTIR end-station at ID21 was used to monitor changes in protein conformation as well as in the overall protein and lipid contents in the proximity of tattoo particles. Synchrotron μ-FTIR analyses allow the assumption that tattoo pigments became located in a lipid-rich β-sheet protein environment.

**Figure 5:**
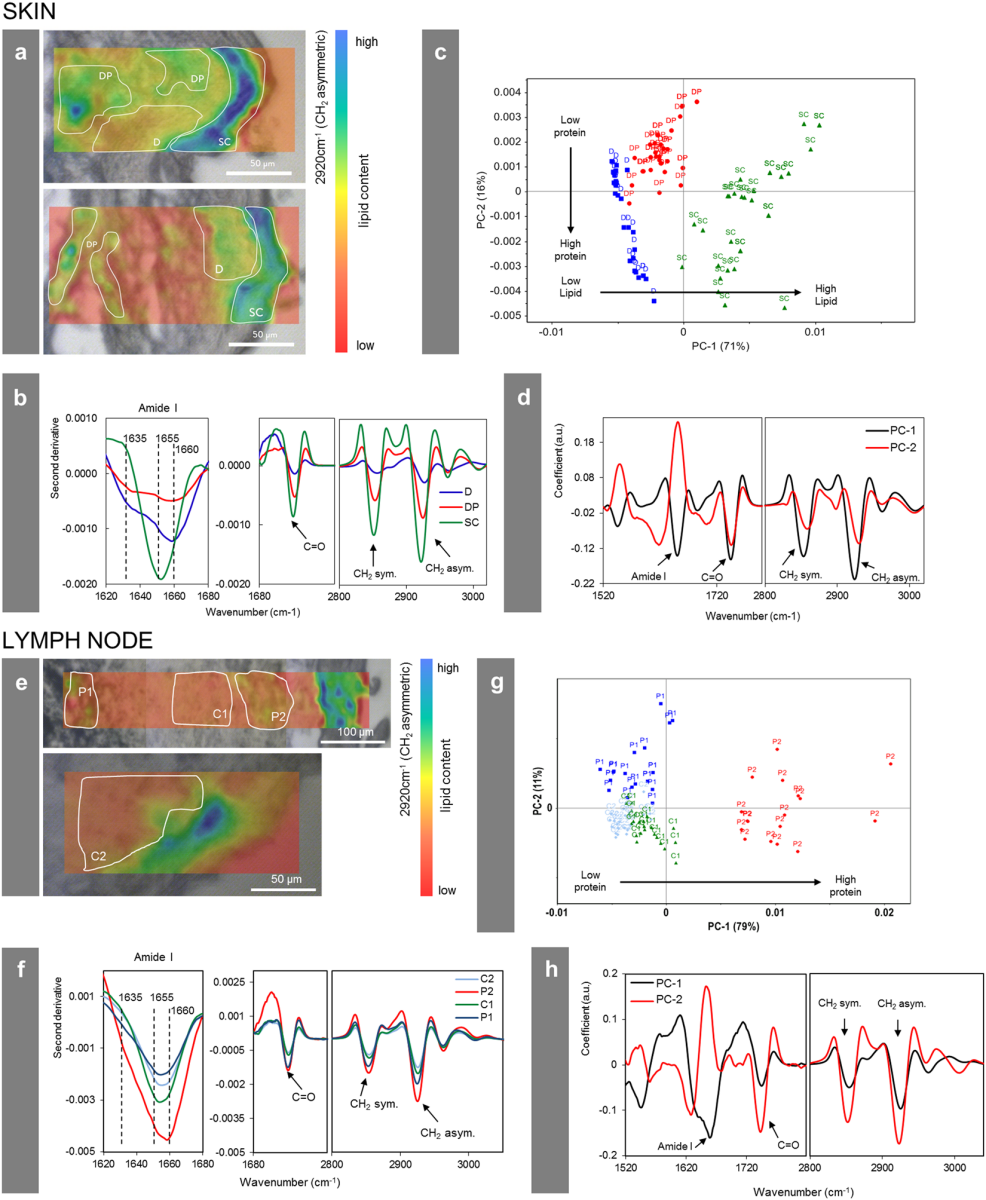
Changes of the biological composition and structure in the cellular proximity of tattoo pigment particles. Section of donor 4 analyzed by means of synchrotron μ-FTIR at ID21, ESRF. (**a**,**e**) Maps in second derivative obtained at 2920 cm^−1^ (–CH_2_ asymmetric vibration) of two different areas in either the skin or lymph node of donor 4 in overlay with a visible light microscopy image. Single points for PCA analysis in (**c**) and (**g**) were picked from the indicated areas. (**b**,**f**) Mean spectra from each region marked in (**a**,**e**) in second derivative. (**c**,**g**) PCA score plot of PC-1 vs. PC-2. (**d**,**h**) Loading plots of PC-1 and PC-2. Abbreviations: SC = *stratum corneum* and epidermis; D = dermis; DP = dermis with particles; P1, P2 = particle-containing regions; C1, C2 = control regions without particles.

The very same sections investigated by means of μ-XRF at ID21 were analyzed by means of μ-FTIR, prior to X-ray analyses, to facilitate exact site matching (cf. Figures 3 and 5). Thus, μ-FTIR results were not altered by μ-XRF radiation of the tissue sections. The high synchrotron photon flux allowed for high spatial resolution. Accordingly, the beam and pixel sizes were reduced to 10 × 10 µm² and 8 × 8 µm², respectively. This resolution is sufficient to distinguish regular dermis from pigment containing areas in the dermis, but remains insufficient to unambiguously separate the *stratum corneum* from the epidermis, which were analyzed here as a single domain (see below). Specific spectral changes related to the modification of biomolecule composition and conformation are displayed using donor 4 as an example, on the basis of two μ-FTIR maps obtained in a single section at two different locations, for the skin and regional lymph node (Fig. 5). The absorption band which peaks at 2920 cm^−1^ corresponds to the *–*CH_2_ stretching mode, which is much more intense in lipids than in proteins. It can be used to qualitatively map the distribution of lipids over thin sections (Fig. 5a). It shows a higher intensity in the *stratum corneum*, as expected^33^. These maps also qualitatively show a higher intensity in the areas of dermis containing tattoo pigments compared to pigment-free control regions. Based on the microscopic images and the μ-XRF maps described earlier, three regions were selected on each map. For the skin section, we divided the obtained map into *stratum corneum* and epidermis (SC), dermis without pigment (D) and dermis around pigment particles (DP) (Fig. 5a). Spectra in the second derivative of these areas were statistically analyzed by means of Principal Component Analysis (PCA). Distribution of points along the PC-1 axis confirms that D and DP have fewer lipid-related long alkyl chains (*–*CH_2_ stretching mode, asym. at 2920 cm^−1^ and *–*CH_2_ sym. at 2854 cm^−1^) and ester (*–*C = O stretching mode, peak at 1745 cm^−1^) vibrations than SC, and that DP regions contain higher levels of lipids than D (Fig. 5b–d). PC-2 separates DP from D and SC since the latter two have higher protein concentrations.

In addition to determining the component distribution, μ-FTIR can be used to identify and map the protein secondary structures across skin sections^33^. In the epidermis, keratinocytes differentiate to finally form the dead, protein- and lipid-rich *stratum corneum*. In the designated SC area of our investigation—comprising also the epidermal layer—the amide I peak maximum at ~1655 cm^−1^ (Fig. 5b) derives from α-helices present in keratin^33^. In the dermis, the peak maximum located at 1660 cm^−1^ corresponds to triple helices present in collagen, while the β-sheet shoulder at 1635 cm^−1^ can be assigned to crosslinked collagen fibers^33^. In the proximity of the pigment particles (DP), the protein content is lower compared to other parts of the collagen-rich dermis. However, the β-sheet shoulder at 1635 cm^−1^ becomes more pronounced close to the particles (Fig. 5b). The *–*CH_2_ and *–*C = O vibrations related to lipids are also higher in the proximity of particles compared to other parts of the dermis. Both findings suggest the presence of denatured β-sheet-rich protein and lipid membranes surrounding the pigment particles. Other investigations have shown that when in contact with foreign surfaces, protein structures can be altered towards the formation of β-sheets^34^. In the skin of donor 2, a similarly enhanced lipid content and the presence of β-sheet structures in the dermis around particles were also noticed (see Supplementary Fig. S4). A statistical comparison of particle-containing and particle-free areas in the lymph node tissue of donor 4 showed a similar increase of lipid contents in the former (Fig. 5e–h). However, no consistent differences in the kind of protein folding could be observed in lymph nodes.

## Discussion

In this investigation, we found a broad range of tattoo pigment particles with up to several micrometers in size in human skin but only smaller (nano)particles transported to the lymph nodes. The exact size limit preventing this translocation is unknown yet. The deposit of particles leads to chronic enlargement of the respective lymph node and lifelong exposure. With the detection of the same organic pigments and inorganic TiO_2_ in skin and lymph nodes, we provide strong analytical evidence for the migration of pigments from the skin towards regional lymph nodes in humans. So far, this only has been assumed to occur based on limited data from mice and visual observations in humans^13, 35^. We also were able to prove the presence of several toxic elements, such as Cr and Ni, derived from tattooing. However, elemental deposits in lymph nodes which were not found in the corresponding skin revealed that tattooing might not have been the only route of exposure in these particular individuals whose tissues were removed after their demise.

It is known that pigments reside in lysosomes or stay attached to membranes of dermal fibroblasts^8, 36^, an observation that supports our μ-FTIR findings of concentrated lipid levels in the proximity of pigment particles. Long alkyl chains and ester groups which we assigned to lipids may also derive from components of tattoo inks, e.g. thickening polymers, surfactants and pigment coatings. However, the frequently used polyethylene glycol^37^ and polyvinyl pyrrolidone polymers below 20 kDa^38^ are known to be metabolized and secreted. In addition, the strong lysosomal and reactive oxygen species-driven reaction of macrophages against foreign material was shown to alter even the highly stable polyurethane^39^. We therefore assume these additives being biodegradable *in vivo* and thus not anymore present in healed tattooed skin. Since the initially used coatings and surfactants of the particles were unknown, interferences in μ-FTIR cannot be fully excluded though.

In cases where foreign hydrophobic material is introduced into the body, fibrinogen and other proteins are likely to become adsorbed and denatured at its surface, thus leading to the generation and presentation of pro-inflammatory matter and subsequent recruitment of immune cells as the initial step in the triggered foreign body reaction^40, 41^. This assumption becomes supported by our μ-FTIR data on β-sheet associated conformational changes of proteins in the proximity of hydrophobic, insoluble tattoo pigments. Foreign body reactions are known from subcutaneous injections of TiO_2_^42^. Despite the hydrophobic nature of pigment surfaces and the here confirmed β-sheet protein conformation in the proximity of tattoo pigments in skin, most tattooed individuals including the donors analyzed here do not suffer from chronic inflammation though. Yet, granulomatous foreign material reactions are among the main non-infectious side effects occurring upon tattooing^43^. Factors preventing the progression towards adverse foreign body reactions in most tattooed individuals despite a β-sheet conformation need to be further investigated.

In future experiments we will also look into the pigment and heavy metal burden of other, more distant internal organs and tissues in order to track any possible biodistribution of tattoo ink ingredients throughout the body. The outcome of these investigations not only will be helpful in the assessment of the health risks associated with tattooing but also in the judgment of other exposures such as, e.g., the entrance of TiO_2_ nanoparticles present in cosmetics at the site of damaged skin.

## Methods

### Human sample preparation

Samples of tattooed skin and regional lymph nodes as well as skin and lymph node samples of two additional donors without any tattoos were taken *postmortem* at the Institute of Forensic Medicine at the Ludwig-Maximilians University of Munich (court-ordered autopsies without any additional cosmetic impairment to the skin). The experiments were performed according to the Helsinki Declaration of 1975. All samples were obtained anonymously without information on the patients disease status, causes of death or demographies. Ethical approval of human biopsy samples was granted by the Ethics committee of the Medical Faculty of the Ludwig-Maximilians University of Munich. We selected specimens with tattoos other than black and which are more likely to contain TiO_2_ and organic pigments. The sample size was limited by the availability of specimens and the beamtime at ESRF. Tissue samples were stored in plastic bags at −20 °C directly after excision and further processed for analysis within a year. Subsamples were cut using a standard scalpel and frozen in TissueTek O.C.T. matrix (Sakura Finetek, Staufen, Germany) for cryo-microtome sectioning. Sections of 5 or 6 µm were obtained and mounted on BaF_2_ substrates (Crystal GmbH, Berlin, Germany) for μ-FTIR and μ-XRF measurements at ID21. Sections for fluorescence light microscopy had a thickness of 6–10 µm and were deposited on standard glass slides, while ν-XRF analyses at ID16B were performed on 12–14 µm sections on 4 µm Ultralene window films (Spex Sample Prep, Metuchen, NJ, USA) mounted on Si_3_N_4_ windows. Sections were inactivated using 4% formaldehyde buffer for 10 min and subsequently washed with deionized water (2 times, 2–5 min). For μ-FTIR and μ-XRF analyses, samples were freeze-dried and stored in a dehydrated environment. Sections on microscopic glass slides were mounted in DAPI-Fluoromount G (Southern Biotech, Birmingham, AL, USA) for cell nucleus staining.

### ICP-MS analysis

Elemental compositions of in total 20 skin and 25 lymph node samples of tattooed donors as well as 2 skin and 2 lymph node samples of non-tattooed donors were analyzed using a nitric acid microwave digestion (Ultraclave, MLS, Leutkirch, Germany). Samples were directly adjacent to those used in other parts in this investigation. Five milliliter of 69% nitric acid was added to 50–200 mg tissue samples in Teflon vessels and heated in the microwave with the following steps: 20–80 °C (3.5 min, 100 bar, 700 W); 80–130 °C (10 min, 120 bar, 1000 W); 130–200 °C (6.5 min, 150 bar, 1000 W), 200 °C (30 min, 150 bar, 1000 W). Elemental concentrations given in ppm are calculated in relation to the weight of digested tissue. Nitric acid was purified using a duoPUR quartz sub-boiling distillation system (MLS, Leutkirch, Germany). Ultrapure water was obtained using a Milli-Q Advantage A10 water purification system equipped with a Millipore Q-POD Element Unit (both from Merck, Darmstadt, Germany). Standards for ICP were purchased either from Sigma Aldrich (Munich, Germany; i.e. Sc, Al, Cu, Ni, Hg) or Merck (Darmstadt, Germany) in the case of In. For Cr, Fe and Cd 1000 mg/l standard solutions in diluted nitric acid were obtained from VWR (Darmstadt, Germany).

A 20-fold dilution of each sample was prepared including 10 ppb of the elements In and Sc as internal standards. XSeries II ICP-MS (Thermo Fischer Scientific, Bremen, Germany) together with an ESI SC2 autosampler (Elemental Service & Instruments, Mainz, Germany) were used for sample analysis. Sample analysis was carried out in triplicate with 100 sweeps each. Resolution was set to 0.02 amu and the dwell time for all elements was 10 ms. Measurements were carried out with collision cell in either −3.0 V mode (In, Sc, Cr, Fe, Ni, Cu, Cd) or 0.0 V mode (Sc, Al). H_2_/He (7% v/v) was used as the collision gas with 5 ml/min flow rate. Data were processed with PlasmaLab 2.5.11.321 (Thermo Scientific, Bremen, Germany).

### LDI-ToF-MS identification of organic pigments

In total 8 skin and 8 lymph node samples of tattooed donors as well as 2 skin and 2 lymph node samples of non-tattooed donors were analyzed. Samples between 50–200 µg were lysed using 1 mg/ml collagenase from *Clostridium histolyticum* Type IA (Sigma Aldrich, Munich, Germany) with an incubation time of at least 24 hours at 37 °C. Lysates were heat-inactivated at 90–95 °C for at least 12 hours. Precipitated pigment particles were washed twice with PBS. Centrifugation was carried out with 500× g for 10 min. Precipitates were applied as thin films to a ground steel target plate with a plastic pipette tip and measured using an UltrafleXtreme MALDI-ToF/ToF (Bruker Daltonik, Bremen, Germany). Spectra were obtained by averaging 3000 individual spectra, with a laser rate of 1000 Hz in positive reflector mode. The instrument was calibrated prior to each measurement with an external ProteoMass™ MALDI Calibration Kit (Sigma Aldrich, Munich, Germany). Data were processed using the flexControl 3.4 and flexAnalysis 3.4 software (Bruker Daltonik, Bremen, Germany).

### Synchrotron FTIR microscopy

FTIR microscopy analyses were performed at beamline ID21 at the European Synchrotron Radiation Facility (ESRF) in Grenoble, France^44^. The beamline is equipped with a Thermo Nicolet Continuum (Thermo Scientific, Madison, WT, USA) microscope coupled to a Thermo Nicolet Nexus FTIR spectrometer (Thermo Scientific, Madison, WT, USA) with a 32x objective, a motorized sample stage, and a liquid nitrogen-cooled 50 µm HgCdTe detector. Maps were acquired in transmission mode using a 10 × 10 µm² beam, step size of 8 µm. Spectra were recorded as an average of 64 scans per spectrum, over a range of 4000 to 850 cm^−1^ and with a spectral resolution of 4 cm^−1^.

The OMNIC software was used to transform spectra from maps of skin and lymph node samples to second derivatives using Savitsky-Golay of second polynomial order with 21 smoothing points^45, 46^. Unscrambler X software (Version 10.3, CAMO Software, Oslo, Norway) was used for further statistical analysis. Principal component analysis (PCA) was performed on the mean-centered data using the spectral regions from: 1800 to 1350 cm^−1^ (related to proteins) and 3200 to 2800 cm^−1^ (related to lipids)^47, 48^. PCA was performed separately for skin and lymph node samples. Score plots and loading plots obtained by PCA analysis as well as mean values from the regions of interest were used for data interpretation.

### Synchrotron μ-XRF and μ-XANES

μ-XRF and μ-XANES analyses were carried out at the beamline ID21^49^. Here, X-rays were generated by an U42 undulator operated in “gap-tracking” mode, i.e. the gap value was optimized for each energy. A fixed exit double-crystal Si(111) Kohzu-monochromator was used in combination with a Ni-coated flat double-mirror rejecting high-energy harmonics and allowed for energy selection with about 0.4 eV resolution of the primary radiation at Ti K-edge (5.1 keV). Downstream of the monochromator, the beam was focused down to 0.4 × 0.8 µm^2^ (vertical × horizontal) using a fixed-curvature Kirkpatrick-Baez (KB) mirror system. The flux was 1.6 × 10^10^ photons/s (~180 mA SR current in multi-bunch mode). A 30 µm Al attenuator was used to reduce the photon flux by one order of magnitude to keep the XRF detector dead time within its linear range. A photodiode collecting the XRF from a thin Si_3_N_4_ membrane inserted in the beam path was used to continuously monitor the incoming beam intensity. XRF and scattered radiation were collected with a dispersive energy silicon drift detector with an active area of 80 mm² (Bruker Daltonik, Bremen, Germany). Acquisition time per point was 100 ms. The pixel size for collecting the XRF maps was adjusted to the regions of interest and varied from 0.5 µm to 5 µm. Scans were performed in continuous (zap) mode and an energy of 5.05 keV was selected for μ-XRF mapping. For collecting Ti XANES spectra, the energy of the incoming beam was scanned from 4.95 to 5.1 keV in increments of 0.5 eV, with acquisition times of 100 ms per energy. Depending on the concentration of the probed region, between 1 and 10 μ-XANES spectra were collected per point and subsequently averaged. Full-field XANES maps were also collected to total the XANES spectra over multiple pixels.

### Synchrotron ν-XRF

The analysis on an adjacent section of skin and lymph node tissue from donor 4 was performed by means of ν-XRF at ID16B at the ESRF. The experimental set-up is described elsewhere^50^. A pink beam with an energy of 17.5 keV with ΔE/E = 1% was focused down to 50 × 50 nm² using KB mirrors. The flux of >1 × 10^11^ photons/s was subsequently reduced using gold and silicon attenuators to keep the dead time on the XRF detectors within the linear range. Two three-element silicon drift detector arrays (SGX Sensortech, Buckinghamshire, UK) were used. The two ν-XRF maps were recorded with a step size of 50 × 50 nm² and 100 ms dwell time. In contrast to the set-up installed at ID21, ID16B operates in air. For estimating the particle size of TiO_2_, analysis was performed on 10 particles by computing the full width at half maximum of line profiles through the particles.

### Availability of materials and data

XRF and FTIR data sets can be provided by the authors upon individual request.

### Ethical approval of human biopsy samples

Samples of tattooed skin and regional lymph nodes were taken *postmortem* and anonymously at the Institute of Forensic Medicine at the Ludwig-Maximilians University of Munich in the frame of court-ordered autopsies without information on the patients disease status, causes of death or demographies. Experiments were performed according to the Helsinki Declaration of 1975 (see: http://www.wma.net/en/30publications/10policies/b3/17c.pdf). Ethical approval of human biopsy retrieval was granted by the Ethics committee of the Medical Faculty of the Ludwig-Maximilians University of Munich, Germany (confirmation by R.P., member of the ethics committee).

## Electronic supplementary material

Supplementary Information
