# Supplementary material for: Synchrotron-based ν-XRF mapping and μ-FTIR microscopy enable to look into the fate and effects of tattoo pigments in human skin
Source: Sci Rep. 2017 Sep 12;7:11395. doi: 10.1038/s41598-017-11721-z (PMC5595966; doi:10.1038/s41598-017-11721-z)

**Supplementary Data:**

**Synchrotron-based ν-XRF mapping and μ-FTIR microscopy enable to look into the fate and effects of tattoo pigments in human skin**

Ines Schreiver^1,†^, Bernhard Hesse^2,†^, Christian Seim^3^, Hiram Castillo-Michel^2^, Julie Villanova^2^, Peter Laux^1^, Nadine Dreiack^1^, Randolf Penning^4^, Remi Tucoulou^2^, Marine Cotte^2^ & Andreas Luch^1,*^

^1^German Federal Institute for Risk Assessment (BfR), Department of Chemical and Product Safety, Max-Dohrn-Strasse 8-10, 10589 Berlin, Germany

^2^European Synchrotron Radiation Facility (ESRF), 38043 Grenoble, Cedex 9, France

^3^Physikalisch-Technische Bundesanstalt, Department of Temperature and Synchrotron Radiation, Abbestrasse 2-12, 10587 Berlin, Germany

^4^Institute of Forensic Medicine, Ludwig-Maximilians University, Munich, Germany

^†^These authors contributed equally to this work

*Correspondence: Andreas Luch, German Federal Institute for Risk Assessment (BfR), Department of Chemical and Product Safety, Max-Dohrn-Strasse 8-10, 10589 Berlin, Germany. Tel.: +49 30 18412 4538. E-mail: andreas.luch@bfr.bund.de

**Supplementary Table S1.** Organic pigments in human skin and lymph node samples from additional donors analyzed by LDI-ToF-MS.

| **Donor** | **Tissue** | **Location** | **Color** | **Pigment** |
| --- | --- | --- | --- | --- |
| Donor 2 | Skin | left | black, red | red 170 (C.I.12475) , blue 15 (C.I.74160) |
|  | LN | left |  | red 170 (C.I.12475) , blue 15 (C.I.74160) |
| Donor 5 | Skin |  | black, red | green 7 (C.I.74260) , red 112 (C.I.12370), blue 15 (C.I.74160) |
|  | LN | hilus | red |  |
|  | LN |  | black | blue 15 (C.I.74160) |
| Donor 6 | Skin | left | green | blue 15 (C.I.74160), green 7 (C.I.74260) |
|  | LN | left | green | green 7 (C.I.74260) |
| Control 2 | Skin | proximal | - | - |
|  | LN | axillary | - | - |

**Supplementary Table S2.** Element concentrations per tissue weight (ppm) in human skin and lymph node samples from additional donors analyzed by ICP-MS.

| **Donor** | **Tissue** | **Location** | **Color** | **Al** | **Cr** | **Fe** | **Ni** | **Cu** | **Cd** | **other^#^** |
| --- | --- | --- | --- | --- | --- | --- | --- | --- | --- | --- |
| 2 | Skin | left | black, red | 2.49 | 9.34 | 95.4 | 0.33 | 6.54 | < LOQ |  |
|  | LN | left |  | 11.3 | 6.77 | 238 | 3.50 | 67.0 | < LOQ |  |
| 5 | Skin |  | black, red | 0.62 | 0.36 | 138 | < LOQ | 1.72 | < LOQ | Rb |
|  | LN | hilus | red | 1.12 | 0.36 | 758 | 0.90 | 2.33 | < LOQ | Ti |
|  | LN |  | black | 0.88 | < LOQ | 193 | < LOQ | 4.33 | 0.69 | Ti, Br, Rb, Hg |
| 6 | Skin | left | green | 4.40 | 0.67 | 76.9 | 0.54 | 19.2 | < LOQ |  |
|  | LN | left | green | 6.19 | 9.29 | 735 | 5.09 | 84.2 | 0.83 |  |
| 7 | LN |  |  | 3.66 | 0.65 | 123 | 0.23 | 3.52 | < LOQ | Ti, Mn, Zn, Rb, Ba |
|  | Skin |  | red, green, black | < LOQ | < LOQ | 4.85 | < LOQ | 2.29 | < LOQ | Ti |
| 8 | Skin | right leg | black | 7.56 | < LOQ | 19.2 | < LOQ | 2.96 | < LOQ | Zn |
|  | LN | right inguinal | black | 2.15 | < LOQ | 21.5 | < LOQ | 0.63 | < LOQ | Zn |
|  | Skin | left leg | black | < LOQ | < LOQ | 8.73 | < LOQ | < LOQ | < LOQ | Zn |
|  | LN | left inguinal | black | 0.16 | 0.06 | 4.65 | < LOQ | 0.10 | < LOQ | Mn |
|  | Skin | right arm | green | 4.11 | < LOQ | 14.8 | < LOQ | 11.5 | < LOQ | Ti, Ba |
|  | Skin | right arm | red | 38.5 | < LOQ | 10.1 | < LOQ | < LOQ | < LOQ | Zn |
|  | LN | right axillary | black | 1.30 | 0.23 | 14.9 | < LOQ | 2.50 | < LOQ | Ti |
|  | Skin | left arm | red, yellow, orange | 5.17 | < LOQ | 5.66 | < LOQ | < LOQ | < LOQ | Zn |
|  | LN | left axillary |  | 1.75 | < LOQ | 14.5 | < LOQ | 0.51 | 0.15 | Mn, Zn, Rb |
|  | LN | trachea |  | 20.8 | < LOQ | 63.0 | < LOQ | 1.28 | < LOQ | Mn, Ba |
| 9 | Skin | left arm | green | 6.49 | < LOQ | 3.29 | < LOQ | 2.03 | < LOQ | Ti, Zr |
|  | LN | left axillary | black | 7.29 | < LOQ | 26.2 | < LOQ | 0.80 | < LOQ | Ti, Mn, Zn, Rb, Ba |
| 10 | Skin | leg | red | 8.24 | < LOQ | 4.26 | < LOQ | < LOQ | < LOQ | I |
|  | LN | inguinal |  | 3.51 | < LOQ | 12.9 | < LOQ | 0.83 | < LOQ | Ti, Mn, Zn, I |
|  | LN | trachea |  | 4.10 | < LOQ | 76.7 | < LOQ | 1.41 | < LOQ | Mn, Zn, I |
|  | LN | hilus |  | 0.85 | < LOQ | 103 | < LOQ | 3.14 | < LOQ | Mn, Zn, Rb, I, Ba, Pb |
|  | LN | para aortic |  | 4.20 | < LOQ | 37.7 | < LOQ | 1.23 | < LOQ | Zn, I |
| 11 | Skin | right arm | green, blue | < LOQ | < LOQ | 2.36 | < LOQ | < LOQ | < LOQ | Zn, Ba |
|  | LN | right axillary | black | 7.55 | 4.68 | 89.4 | < LOQ | 5.33 | 36.4 | Ti, Ba, Hg |
| 12 | Skin | left | red, green, black | < LOQ | < LOQ | 49.5 | < LOQ | 1.66 | < LOQ |  |
|  | Skin | left | red, green, black | < LOQ | < LOQ | 29.9 | < LOQ | 1.42 | < LOQ | Zn |
|  | LN | left | black | < LOQ | < LOQ | 129 | < LOQ | 0.86 | < LOQ | Zn |
| 13 | Skin | right leg | back, blue | 38.6 | 1.76 | 94.6 | 0.73 | 28.1 | < LOQ | Ti, Mn, Zn, Ba |
|  | LN | right inguinal |  | 5.80 | 0.71 | 200 | 0.37 | 12.5 | < LOQ | Ti, Mn, Zn, Cd, Sn |
|  | Skin | left arm | black, red | 3.35 | 0.96 | 207 | < LOQ | 2.99 | < LOQ | Mn, Zn |
|  | LN | left axillary |  | 1.43 | 1.56 | 279 | 0.72 | 3.75 | < LOQ | Ti, Mn, Zn, Cd, Ba |
|  | LN | coeliac |  | < LOQ | < LOQ | 155 | < LOQ | 3.26 | < LOQ | Ti, Mn, Zn, Cd |
|  | LN | left para aortic |  | < LOQ | < LOQ | 567 | < LOQ | 4.67 | < LOQ | Ti, Mn, Zn, Cd |
|  |  |  | Average | 8.23 | 1.42 | 80.1 | 0.61 | 4.06 | 18.3 |  |

Abbreviations: LN = lymph node; LOQ = limit of quantification. Elements measured (non-specified oxidation states): aluminum (Al), barium (Ba), bromine (Br), cadmium (Cd), chromium (Cr), copper (Cu), iodine (I), iron (Fe), lead (Pd), manganese (Mn), mercury (Hg), nickel (Ni), rubidium (Rb), selenium (Sn), titanium (Ti), tungsten (W), and zinc (Zn).

^#^Non-quantitatively identified elements.

**Supplementary Figure S1.** Identification of the used organic colorants by LDI-ToF-MS spectrometry. **a-e**) Mass spectra obtained from lysed skin or lymph node specimens are displayed next to spectra from pure reference pigments and their calculated isotope distribution (cf. Fig. 2).

**
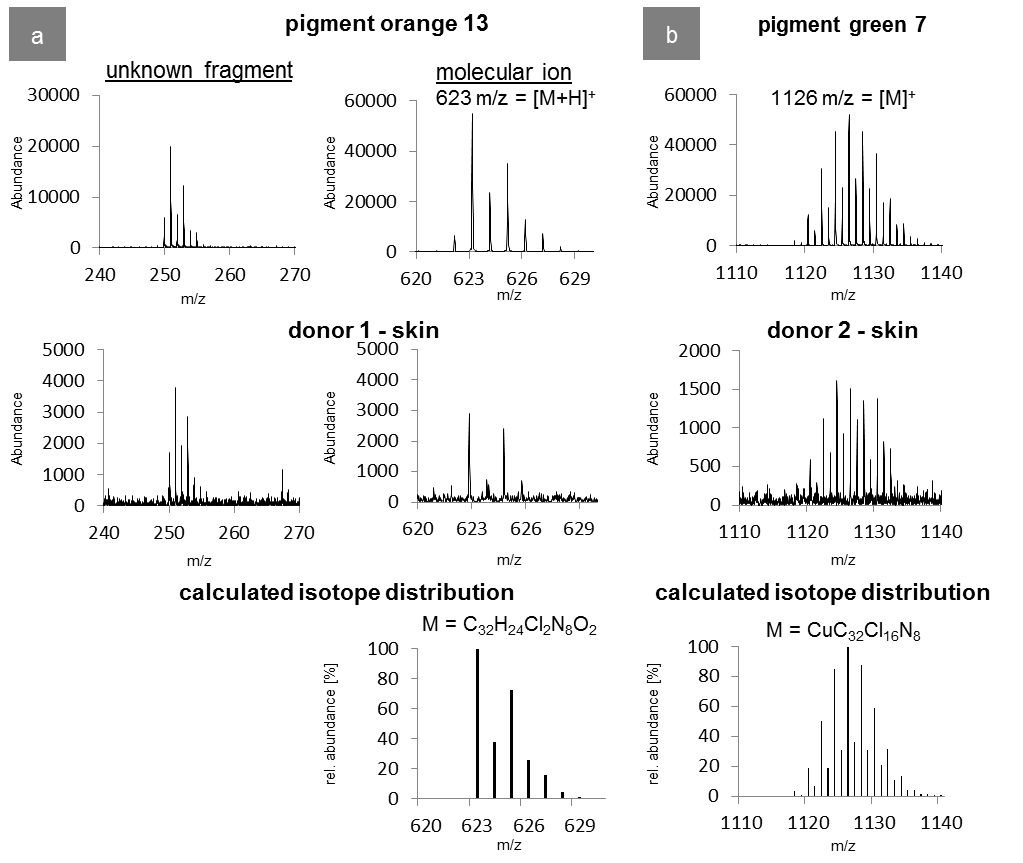
**

**
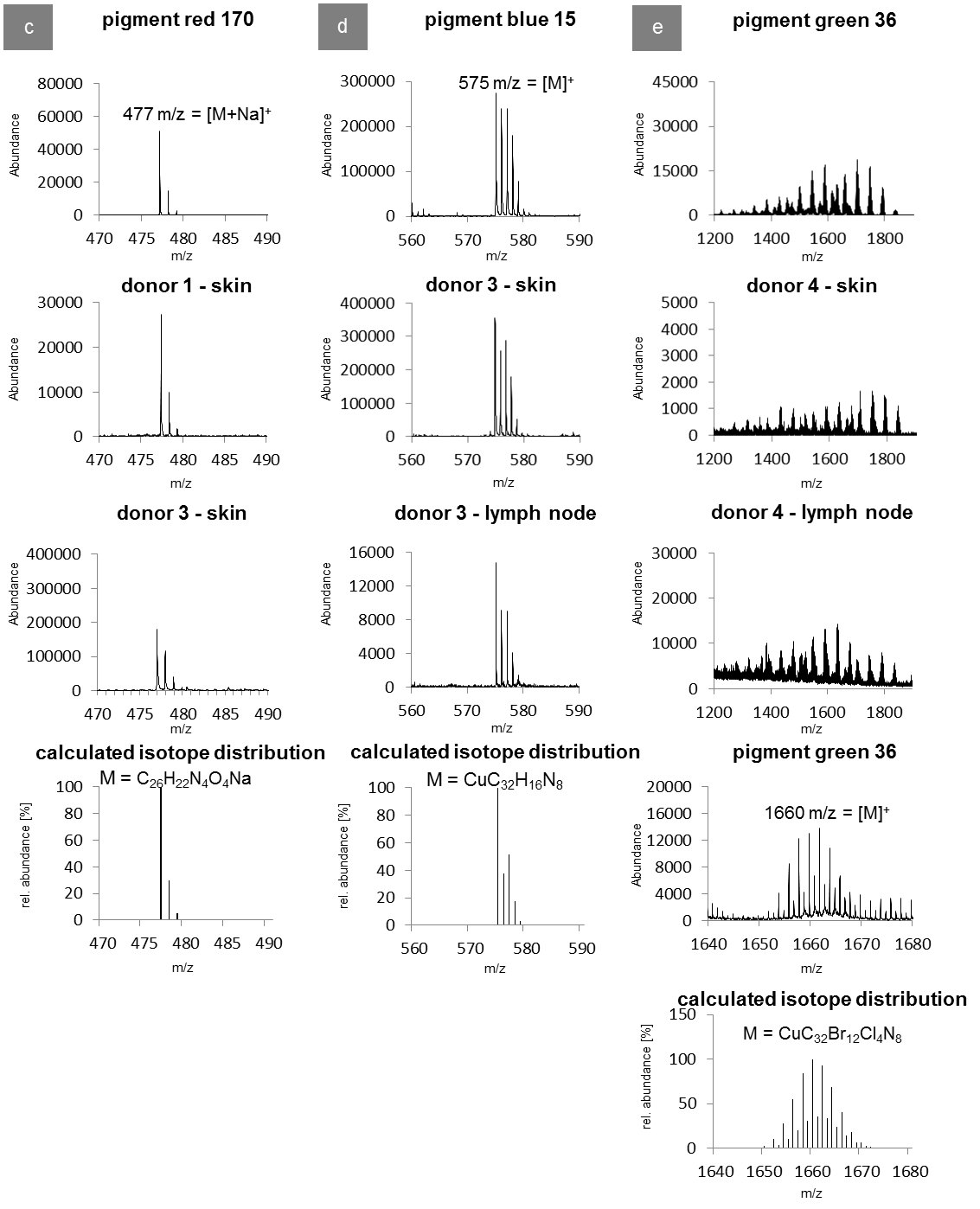
**

**Supplementary Figure S2.** μ-XRF mapping links elements to tattoo particles. Sections of skin and lymph node tissues of donors 1 and 3 were analyzed by μ-XRF at ID21, ESRF. **a,** **d**) Optical microscopy images of adjacent sections. **b, e**) μ-XRF maps of tissue sections. In skin, the epidermal layer is visualized by the elements P (cell nuclei, blue) and the *stratum corneum* by S (protein, green). The lymph node capsule of donor 1 shows also high S content (green layer, right). Cl (green) is increased in the proximity of Ti (red) in the dermal layer of donor 1 (left image) and 3 (right image). High Ti in the lymph node of donor 3 led to detector saturation. **f**) High resolution map of skin in e). Cl and Ti are in close proximity to P-rich cell nuclei. **c, g**) Ti K-edge μ-XANES spectra of skin (donors 1 and 3) and lymph node (donor 3) show mostly rutile TiO_2_ (cf. Fig. 3). No μ-XANES was obtained for the lymph node of donor 1 since Ti concentrations were too low.

**
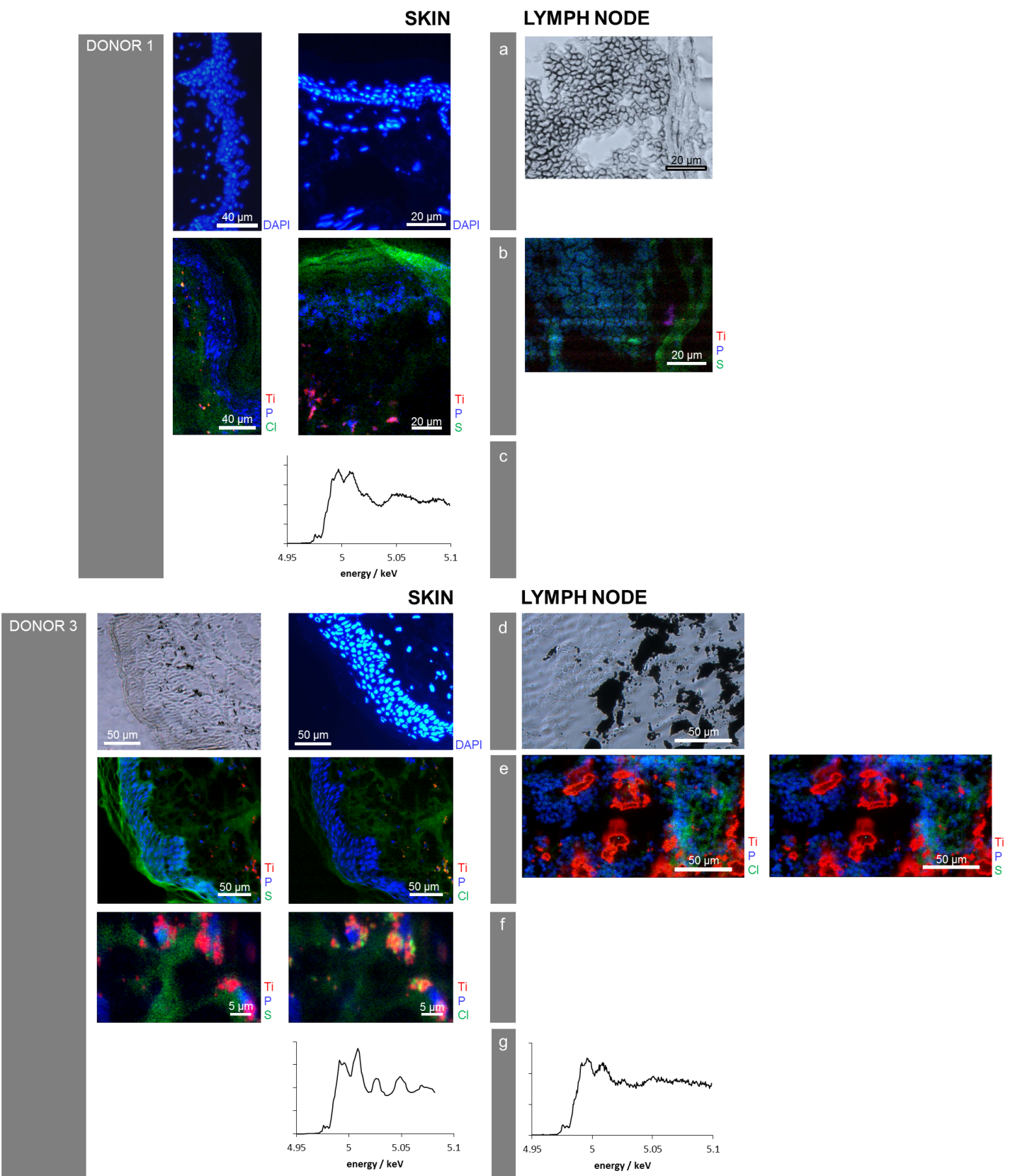
**

**Supplementary Figure S3.** Pigment particles in skin and lymph node tissues of donor 4 explored by using ν-XRF at ID16B, ESRF. **a**) Fe elemental map (log scale). No Fe particles were detected in the skin of donor 4. Dissolved and protein-bound Fe concentrations are higher in cells when compared to the extracellular matrix, allowing the localization of the epidermal layer (arrow) and cells in the proximity of particles in the dermis. **b**) Ti, Cu, Br mapping in the exact same area as displayed in **a**). Br (green) systematically overlays with Cu (blue) to give a turquoise shade in the images, thereby supporting the findings of the brominated copper phthalocyanine pigment green 36. Fe particles in the lymph node **a**) are co-localized to TiO_2_ (red) and pigment green 36 (Cu, Br) particles. Scale bars = 5 µm.

**
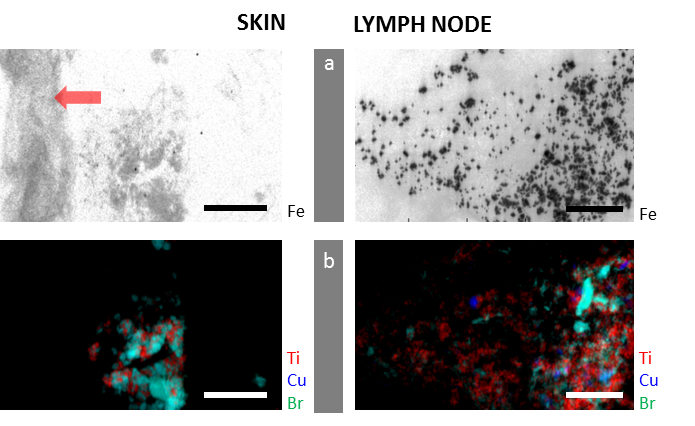
**

**Supplementary Figure S4.** Changes of the biological structures in the cellular proximity of tattoo pigments. Skin section of donor 2 analyzed by means of synchrotron μ-FTIR at ID21, ESRF. **a**) Maps in second derivative obtained at 2920 cm^-1^ (―CH_2_ asymmetric vibration in overlay with the FTIR visible light microscopic picture). Single points for PCA analysis in c) were picked from the indicated areas. **b**) Mean spectra from each region marked in a) in second derivative. Lipid and β-sheet related vibrations were increased in DP compared to D (see text). Similar to donor 4 (cf. Fig. 4), amide I band again separates DP (protein low) from D (protein high). In contrast to the skin of donor 4, the mean spectrum of the D area shows an amide I maximum at 1655 cm^-1^ comparable to the SC area. **c**) PCA score plot of PC-1 vs. PC-2. **d**) Loading plots of PC-1 and PC-2. Abbrevations: SC = *stratum corneum* and epidermis, D = dermis, DP = dermis with particles.


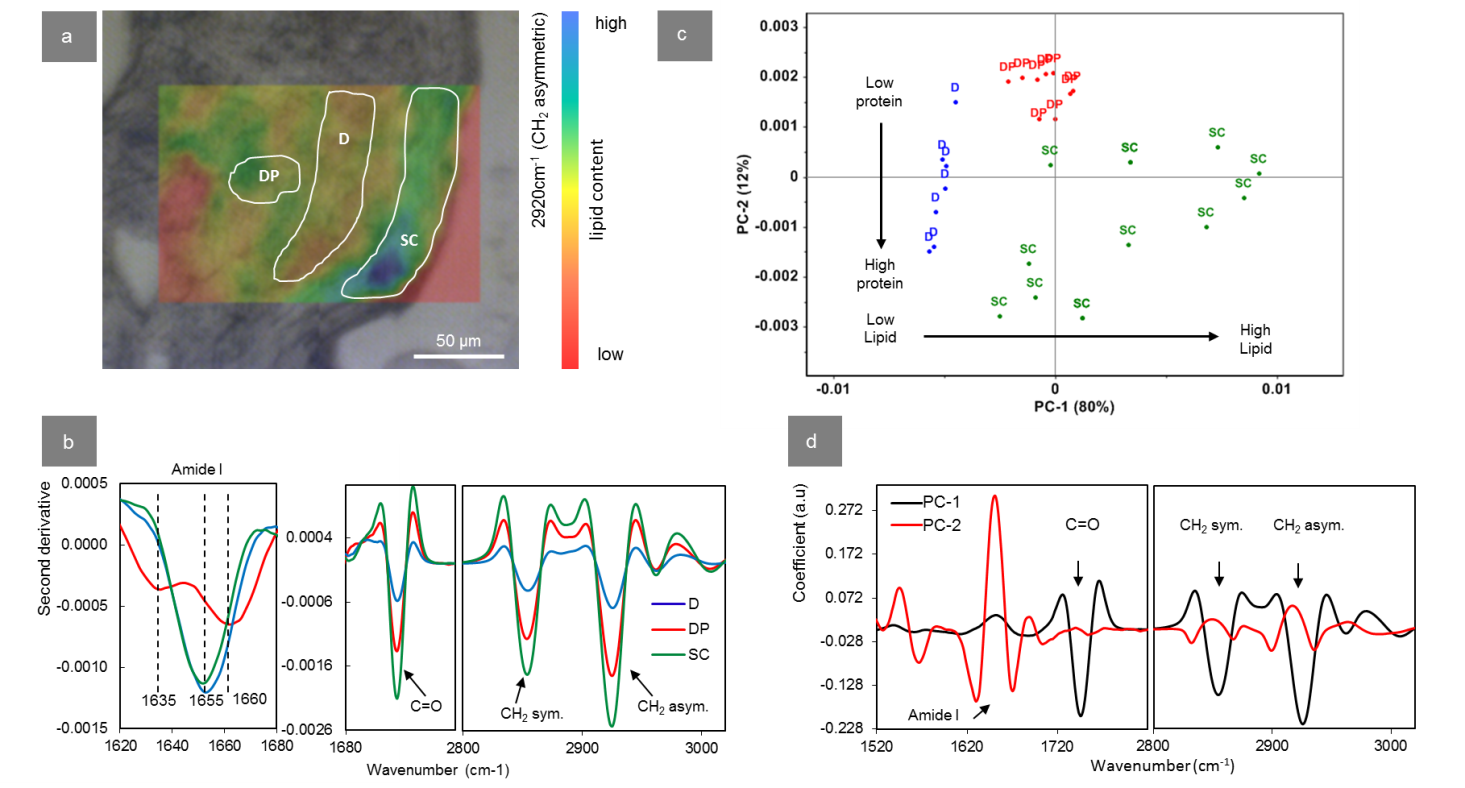

Supplement: Supplementary file 1 — Supplementary Information [file 41598_2017_11721_MOESM1_ESM.doc]
